# Reprogramming brain-resident macrophages: from disease drivers to therapeutic allies in neurological disorders

**DOI:** 10.3389/fncel.2026.1726194

**Published:** 2026-02-09

**Authors:** Peng Zhao, Su-Yi Li, Qun Liu, Xiao-Chun Peng, Lian Liu, Fu-Yuan Yang, Chao Wang, Feng Qian, Feng-Ru Tang

**Affiliations:** 1 Department of Physiology, School of Basic Medicine, Health Science Center, Yangtze University, Jingzhou, Hubei, China; 2 Department of Medical Laboratory, School of Basic Medicine, Health Science Center, Yangtze University, Jingzhou, Hubei, China; 3 Department of Radiology, Jingzhou Hospital Affiliated to Yangtze University, Health Science Center, Yangtze University, Jingzhou, Hubei, China; 4 Department of Pathophysiology, School of Basic Medicine, Health Science Center, Yangtze University, Jingzhou, Hubei, China; 5 Department of Pharmacology, Jingzhou Hospital Affiliated to Yangtze University, Health Science Center, Yangtze University, Jingzhou, Hubei, China; 6 Radiation Physiology Laboratory, Singapore Nuclear Research and Safety Institute, National University of Singapore, Singapore, Singapore

**Keywords:** border-associated macrophage, brain injury, brain-resident macrophage, microglia, neurodegenerative disease

## Abstract

Brain-resident macrophages (BRMs), including microglia and border-associated macrophages (BAMs), are the core immune sentinels of the central nervous system (CNS). They originate from early embryonic yolk sac and fetal liver progenitors and maintain their population throughout life via self-renewal. During neurodevelopment, microglia maintain neural network homeostasis by phagocytosing apoptotic neural precursors and pruning synaptic connections. In adulthood, they rapidly respond to infection, injury, or protein aggregation, which can both promote repair and exacerbate neurotoxicity. BAMs, located in the meninges, perivascular spaces, and choroid plexus, play a key role in boundary homeostasis and peripheral immune signal surveillance. Recent studies reveal that BRMs exhibit dual roles in Alzheimer’s disease (AD), Parkinson’s disease (PD), Huntington’s disease (HD), multiple sclerosis (MS), as well as ischemic stroke, traumatic brain injury, and radiation-induced brain injury: they can protect neurons by clearing pathological proteins or cellular debris, but persistent inflammatory responses may drive neurodegeneration. In AD, microglia clear Aβ plaques via triggering receptor expressed on myeloid cells 2 (TREM2) and ADGRG1 signaling, while BAMs regulate synaptic damage and cerebrovascular function through CD36-ROS and SPP1 pathways. In PD and HD, BRMs contribute to *α*-synuclein- and mutant huntingtin-related inflammatory responses. In MS, BRMs modulate the pro−/anti-inflammatory balance through antigen presentation and cytokine signaling. Based on these mechanisms, therapeutic strategies targeting BRM functions are emerging, including NLRP3 inflammasome inhibitors, TREM2 agonists, and interventions promoting microglial neuroprotective phenotypes. Future approaches aiming to precisely modulate BRM plasticity and their interactions with the peripheral immune system may transform these immune sentinels from “disease drivers” to “therapeutic allies,” offering novel strategies for treating neurodegenerative diseases and brain injuries.

## 1 Introduction

**Figure 1:**
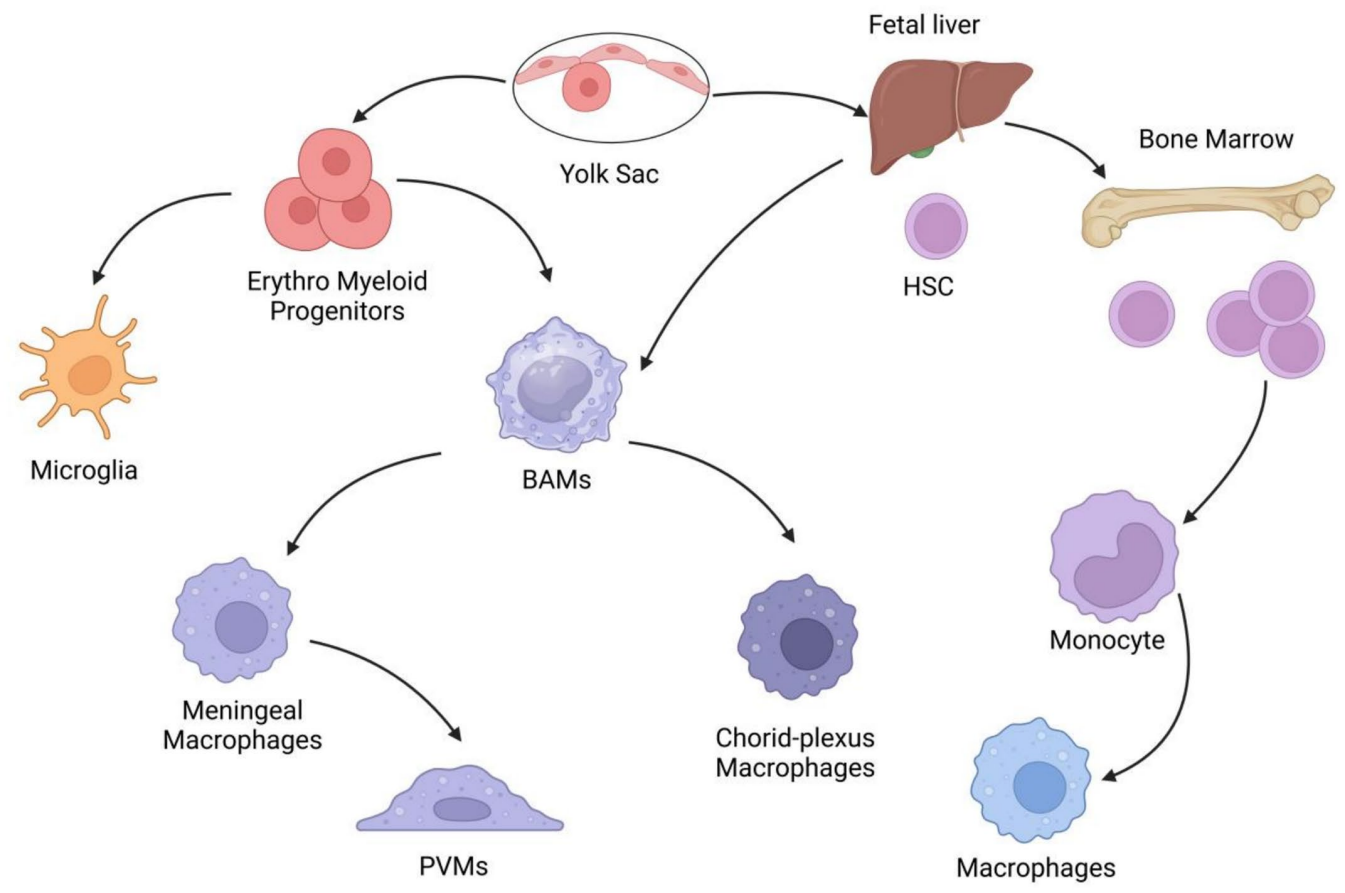
Developmental origins and diversity of brain-resident macrophages (BRMs). Microglia arise from yolk sac-derived erythro-myeloid progenitors (EMPs), migrating to the brain parenchyma during early embryogenesis. Border-associated macrophages (BAMs) originate from primitive macrophages of the yolk sac (early embryogenesis) and hematopoietic stem cells (HSCs) of the fetal liver (later stages), colonizing brain border regions (meninges, perivascular spaces, choroid plexus). Postnatally, BAMs differentiate into three subsets: meningeal macrophages, perivascular macrophages (PVMs), and choroid plexus macrophages, which patrol brain barrier interfaces. Peripheral mononuclear phagocytes (monocytes, dendritic cells) derive from bone marrow HSCs and infiltrate the central nervous system (CNS) under pathological conditions, interacting with BRMs to modulate neuroinflammation and repair.

Macrophages are key sentinels of the innate immune system and a class of myeloid cells capable of phagocytosing pathogens, cellular debris, and toxic particles to maintain tissue integrity. They originate from erythro-myeloid progenitors (EMPs) in the yolk sac, infiltrate tissues during embryonic development, and form tissue-resident macrophages (TRMs) with self-renewal capacity that persist throughout life (Hagemeyer et al., 2016; Lazarov et al., 2023). In the brain, two major TRM populations exist: parenchymal microglia and BAMs located in the border regions of central nervous system (CNS), including the meninges, perivascular spaces, and choroid plexus (Utz et al., 2020; Silvin et al., 2023). During early embryogenesis, primitive macrophages derived from the yolk sac and fetal liver enter the developing brain via the vasculature, colonizing the tissue before the formation of the blood–brain barrier (BBB) and subsequently differentiating into microglia and BAMs (Mosser et al., 2017; Stremmel et al., 2018; Goldmann et al., 2016) (Figure 1). Microglia rely on TGF-*β* signaling and display a CD206-/CX3CR1 + phenotype, whereas BAMs are TGF-β-independent, exhibit a CD206+/CX3CR1- profile, and can be further subdivided into meningeal macrophages, perivascular macrophages (PVMs), and choroid plexus macrophages (CPMs) with distinct developmental origins (Utz et al., 2020; Goldmann et al., 2016; Kierdorf et al., 2019; Van Hove et al., 2019). Postnatally, leptomeningeal macrophages migrate to perivascular niches, mature into PVMs, and maintain their population through clonal expansion (Masuda et al., 2022) (Figure 1). The BBB restricts peripheral immune cell entry into the CNS, ensuring that microglia and BAMs serve as the primary innate immune guardians of the brain (Hoeffel et al., 2015). These brain-resident macrophages (BRMs) “reside” in the tissue and maintain their numbers through self-renewal, with minimal replacement from external sources in adulthood (Viola et al., 2024).

Microglia can adopt distinct functional phenotypes in response to microenvironmental cues, among which the M1 and M2 states represent two prototypical polarization programs. M1 microglia correspond to a classically activated, pro-inflammatory state that is rapidly induced under conditions of infection, injury, or sterile inflammation. Their activation is driven by factors such as IFN-*γ*, TNF-*α*, and cell- or pathogen-associated debris, and is commonly elicited experimentally by stimulation with LPS or IFN-γ (Mills, 2012; Gordon and Taylor, 2005; Martinez and Gordon, 2014). Microglia sense danger signals through a broad array of pattern-recognition receptors (PRRs), including Toll-like receptors (TLRs), NOD/NOD-like receptors (NLRs), and scavenger receptors (SRs), thereby initiating pro-inflammatory responses (Ransohoff and Perry, 2009). At the molecular level, the M1 phenotype is characterized by high expression of pro-inflammatory cytokines such as TNF-α, IL-1β, IL-6, IL-12, and IL-23, as well as inducible nitric oxide synthase (iNOS/NOS2), accompanied by increased production of nitric oxide (NO) and reactive oxygen species (ROS), and upregulation of antigen-presenting and co-stimulatory molecules including MHC-II and CD40 (Hanisch and Kettenmann, 2007; Villalta et al., 2009). In terms of signaling pathways, the inflammatory effects of M1 microglia are closely linked to inflammasome activation, particularly the NLRP3 inflammasome, which promotes caspase-1–mediated maturation and release of IL-1*β* and IL-18, thereby amplifying pro-inflammatory cascades and serving as a key regulatory mechanism in central nervous system inflammation (Martinon et al., 2009; Quintin et al., 2014; Shi et al., 2012). In contrast, M2 microglia represent an alternatively activated state associated with anti-inflammatory responses and tissue repair. Their polarization is primarily induced by IL-4 and IL-13 (M2a), which signal through IL-4Rα to suppress NF-κB–mediated pro-inflammatory pathways and promote resolution of inflammation and tissue repair (Stein et al., 1992; Rutschman et al., 2001). In addition, immune complexes acting through TLR/Fc*γ*R signaling can induce an M2b phenotype, whereas IL-10, transforming growth factor-*β* (TGF-β), and glucocorticoids drive M2c polarization, highlighting the strong context dependence of M2 activation (Mills, 2012). Molecularly, M2 microglia are characterized by high expression of IL-10, IL-13, TGF-β, and markers such as arginase-1 (Arg1), CD206 (Mrc1), Ym1, Fizz1, and PPAR-γ (Colton, 2009; Michelucci et al., 2009). Their core signaling pathways include the IL-10/STAT3 axis and the IL-4/IL-13–STAT6 axis, together with activation of PPAR-γ, which collectively regulate anti-inflammatory responses, phagocytosis of apoptotic cells, and tissue remodeling processes (Lang et al., 2002; Gordon and Martinez, 2010; Rajaram et al., 2010). Overall, microglia exhibit high plasticity and can undergo dynamic functional reprogramming between pro-inflammatory M1 and anti-inflammatory/repair-oriented M2 phenotypes in response to diverse microenvironmental stimuli. This reprogramming, governed by cytokines, danger signals, and integrated metabolic and transcriptional regulatory networks, determines the roles of microglia in neuroinflammation, neuronal injury, and tissue repair, thereby positioning microglial phenotypic modulation as a critical mechanism in central nervous system disease progression and a promising target for therapeutic intervention.

During neurodevelopment, microglia maintain cortical neuronal homeostasis by phagocytosing apoptotic neural progenitors and pruning redundant synaptic connections (Prinz et al., 2021). Additionally, they secrete specific cytokines to regulate neuronal survival and differentiation, contributing to neurogenesis and synaptic circuit formation (Fricker et al., 2018; Shigemoto-Mogami et al., 2014; Teleanu et al., 2022). Microglia, as the resident immune cells of the central nervous system, colonize the brain during early fetal development and actively participate in neurodevelopment by secreting a wide range of cytokines and neurotrophic factors. Under physiological conditions, TGF-*β*, insulin-like growth factor-1 (IGF-1), and brain-derived neurotrophic factor (BDNF) play critical roles in regulating neural progenitor cell proliferation and differentiation, promoting neuronal survival, and orchestrating synapse formation, synaptic pruning, and the maturation of neural circuits. In adulthood, microglia act as dynamic guardians, monitoring the CNS microenvironment. Upon encountering infection, injury, or protein aggregation, they respond rapidly by releasing inflammatory mediators, recruiting peripheral immune cells, and phagocytosing toxic debris (Teleanu et al., 2022; Gao et al., 2023). These responses are critical for restoring homeostasis, but dysregulation can exacerbate neurotoxicity and drive neurodegenerative progression in diseases such as AD, PD, and MS (Teleanu et al., 2022; Gao et al., 2023; Quan and Zhang, 2023; Zhang et al., 2019; Otxoa-De-Amezaga et al., 2019; Strecker et al., 2017).

Neurological disorders often arise from the accumulation of neurotoxic substances (e.g., Aβ plaques, myelin debris), which continuously induce chronic neuroinflammation. Activated BRMs recruit peripheral immune cells, amplifying inflammatory cascades, damaging neurons, and accelerating functional decline (Quan and Zhang, 2023; Zhang et al., 2019; Otxoa-De-Amezaga et al., 2019; Strecker et al., 2017). Paradoxically, BRMs can also suppress excessive inflammation by phagocytosing infiltrating immune cells and their toxic products (Otxoa-De-Amezaga et al., 2019; Zera and Buckwalter, 2020). This duality makes BRMs attractive therapeutic targets. Recent advances in cancer immunotherapy, such as engineered cell therapies and nanoparticle-based drug delivery, have inspired approaches to reprogram microglia and BAMs, aiming to enhance their neuroprotective functions while suppressing harmful responses (Cao et al., 2024).

However, delivering therapies to the CNS remains a significant challenge. Traditional approaches, such as intracerebroventricular or intrathecal injections, bypass the BBB but are highly invasive. Peripheral administration requires advanced delivery systems for CNS specificity, including BBB-penetrating nanoparticles or strategies that transiently modulate BBB permeability (Cao et al., 2024). This review summarizes the multifaceted roles of BRMs in neurological disorders, evaluates emerging therapeutic modulation strategies, and explores the potential of peripheral monocytes as adjuncts for CNS repair. By bridging fundamental neuroimmunology with translational innovations, we highlight approaches to transform these immune sentinels from “disease drivers” into “therapeutic allies.”

## 2 Brain-resident macrophages

Brain-resident macrophages (BRMs) are innate immune cells within the CNS that play critical roles in brain tissue development, homeostasis maintenance, and neuropathological conditions. During early embryogenesis, primitive macrophages from yolk sac and fetal liver progenitors colonize the developing brain via vasculature prior to BBB formation, later differentiating into microglia and BAMs (Mosser et al., 2017; Stremmel et al., 2018; Goldmann et al., 2016) (Figure 1). While microglia depend on TGF-*β* signaling and exhibit a CD206-/CX3CR1 + phenotype, BAMs are TGF-β-independent, display a CD206+/CX3CR1- signature, and comprise heterogeneous subpopulations (meningeal, perivascular, and choroid plexus macrophages) with distinct developmental origins (Utz et al., 2020; Goldmann et al., 2016; Kierdorf et al., 2019; Van Hove et al., 2019). Postnatally, leptomeningeal macrophages migrate into perivascular niches, maturing into PVMs that persist via clonal expansion (Masuda et al., 2022) (Figure 1). These BAMs function as central regulators of neuroimmune signaling at brain barrier interfaces and contribute significantly to the progression of immune-mediated neurological pathologies (Sun and Jiang, 2024) (Figure 1).

According to their location and characteristics, BRMs can be mainly divided into two categories: the first category is parenchymal microglia. Microglia are dynamic regulators of brain health and can respond rapidly to neuronal injury, both promoting repair and potentially exacerbating inflammation (Paolicelli et al., 2022). Under homeostatic conditions, they patrol neuronal somata, axon initial segments, nodes of Ranvier, and synapses, regulating neural circuit structures through direct contact and purinergic signaling (Umpierre and Wu, 2021; Whitelaw et al., 2023; Cserép et al., 2021; Cserép et al., 2020; Colonna and Butovsky, 2017). Specifically, microglia mediate migration and phagocytosis via the purinergic ATP receptor P2Y12 (Cserép et al., 2020; Colonna and Butovsky, 2017); they can shape axo-axonic synapses at the axon initial segment, thereby influencing action potential generation (Gallo et al., 2022); monitor nodes of Ranvier via the KCNK13 potassium channel, coupling neuronal activity with immune surveillance (Ronzano et al., 2021); and prune synapses during development and cognitive processes, optimizing neural network plasticity (Eyo and Molofsky, 2023). In zebrafish models, microglia cooperate with oligodendrocytes to phagocytose abnormal myelin, ensuring the precision of myelination during development (Hughes and Appel, 2020). These functions highlight the central role of microglia in CNS development, cognitive function, and injury repair (Hammond et al., 2019). In addition, numerous studies have shown that microglia play important roles in neurodegenerative diseases. Recent single-cell transcriptomic studies have identified a novel microglial subset closely associated with AD pathology—disease-associated microglia (DAM). DAM formation occurs in two steps: first, microglial checkpoint genes are downregulated via a TREM2-independent pathway, and then full activation is driven by TREM2-dependent signaling, endowing microglia with the ability to clear A*β* and limit neurodegeneration. This finding further emphasizes the core role of microglia and TREM2 signaling in AD pathogenesis and progression (Keren-Shaul et al., 2017). Among these, TREM2 is a key innate immune receptor that is highly expressed on microglia in the central nervous system and plays a central role in regulating microglial survival, phagocytic function, and lipid sensing. By associating with the adaptor protein DAP12, TREM2 activates downstream signaling pathways that maintain microglial viability, metabolic homeostasis, and stress resistance under pathological conditions. In addition, TREM2 facilitates the efficient clearance of apoptotic neurons, amyloid-β aggregates, and myelin debris, thereby limiting secondary neuroinflammation and slowing disease progression. Meanwhile, by recognizing diverse lipid ligands and regulating lipid uptake and metabolism, TREM2 enables microglia to adapt to the lipid-rich environments characteristic of neurodegenerative diseases and to acquire disease-associated phenotypes. Collectively, TREM2 serves as a critical regulator of microglial homeostasis and represents a promising therapeutic target for neurodegenerative diseases, although its modulation must be carefully controlled to avoid excessive inflammatory activation (Li et al., 2025).

The second category is BAMs located in CNS border regions (meninges, perivascular spaces, choroid plexus) (Goldmann et al., 2016; Van Hove et al., 2019), mainly including meningeal macrophages, perivascular macrophages (PVMs), and choroid plexus macrophages (CPMs). They maintain boundary homeostasis and serve as immune sentinels essential for boundary defense. (1) Meningeal macrophages: including dural and leptomeningeal macrophages, patrol the meningeal layers. MHC-II-negative subsets resist neurotropic pathogens (such as LPS, SARS-CoV-2) through interferon-dependent mechanisms, preventing fatal meningitis (Rebejac et al., 2022). With aging, embryonically derived dural macrophages are gradually replaced by bone marrow-derived cells, resulting in impaired meningeal drainage and exacerbated neuroinflammation (Van Hove et al., 2019). (2) PVMs: these cells are distributed around brain blood vessels and detect peripheral invasion signals (such as LPS) earlier than microglia, acting as the first responders to BBB disruption (Kim et al., 2021). They initiate adaptive immune responses in encephalitis through antigen presentation (Qin et al., 2021) and regulate cerebrospinal fluid (CSF) flow by maintaining the integrity of the perivascular matrix (Drieu et al., 2022). During aging, the loss of embryonically derived LYVE1 + PVMs correlates with decreased CSF clearance, suggesting their role in CNS diseases (Siret et al., 2022). PVMs also interact with endothelial cells, smooth muscle cells, astrocytes, and neurons to form the neurovascular unit (NVU). Although endothelial cells dominate BBB function, maintenance of the BBB depends on interactions among vascular-associated cells (Santisteban et al., 2020; Nehra et al., 2022; Denes et al., 2024). The NVU is essential for maintaining CNS homeostasis, regulating cerebral blood flow (CBF), and ensuring selective BBB permeability. Furthermore, PVMs not only maintain BBB integrity but also interact with endothelial cells through the release of vascular endothelial growth factor (VEGF), ROS, pro-inflammatory cytokines, and chemokines (Zheng et al., 2025). (3) CPMs: further subdivided into stromal macrophages (perivascular) and epithelial macrophages (anchored at the blood-CSF barrier). They can migrate to sites of injury to enhance barrier function (Shipley et al., 2020). In hydrocephalus models, their activation promotes CSF production, indicating potential therapeutic value in treating CSF dysregulation (Jin et al., 2021; Robert et al., 2023). Moreover, many studies have shown that BAMs also play important roles in neurodegenerative diseases. In neurodegenerative conditions, BAMs not only contribute to homeostasis but also participate directly in pathological processes. Studies indicate that BAMs are major sources of vascular ROS in the brain, promoting vascular dysfunction and cerebral amyloid angiopathy (CAA) via CD36-mediated signaling. Using Tg2576 AD mouse models, researchers selectively replaced meningeal and perivascular BAMs via bone marrow transplantation, finding that CD36-deficient BAMs significantly suppressed ROS production, restored neurovascular function, and alleviated A*β*1-40-associated CAA, without obvious effects on parenchymal amyloid plaques. More importantly, this intervention improved cognitive function and enhanced vascular clearance of exogenous Aβ. These results directly demonstrate the key role of BAMs in CAA pathogenesis and suggest that they may serve as important therapeutic targets for AD and other CNS disorders (Park et al., 2017).

## 3 Brain-resident macrophages in neurological diseases

### 3.1 Alzheimer’s disease

AD is the most common cause of dementia, defined as the deterioration of cognition, function, and behavior, typically beginning with the loss of recent memory (Lane et al., 2018). The definitive pathological features in the brain tissue of AD patients are extracellular amyloid plaques composed of amyloid-β (Aβ) and intracellular neurofibrillary tangles (NFTs) formed by hyperphosphorylated tau (p-tau) (Winblad et al., 2016). In addition, current therapeutic strategies only improve symptoms and do not effectively treat AD.

**Figure 2:**
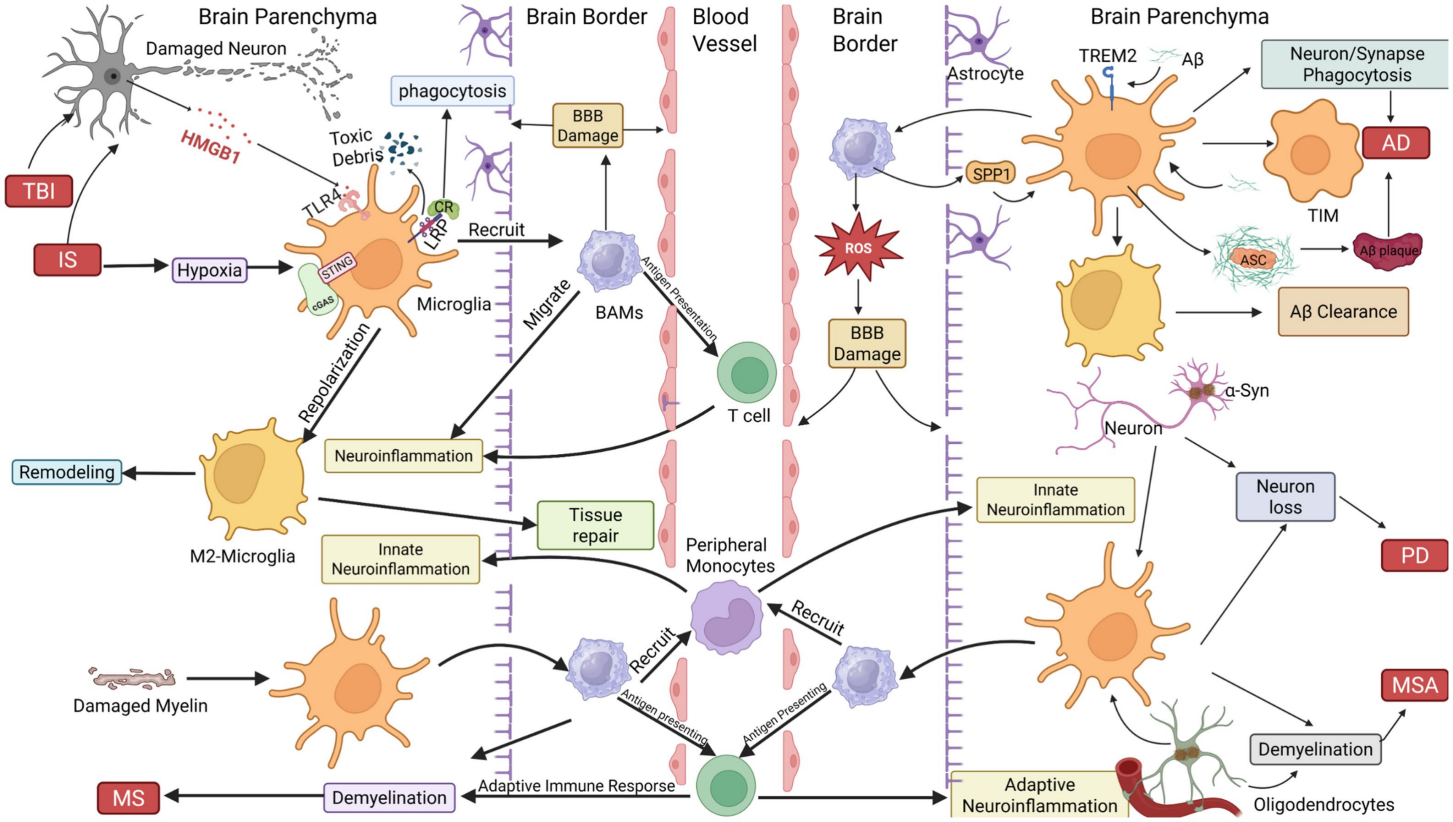
Dual roles of BRMs in neurological disease pathogenesis. (1) Neurodegenerative and acute injury contexts (AD, PD, TBI, IS): Damaged neurons release debris and high-mobility group box 1 (HMGB1), which binds to Toll-like receptor 4 (TLR4) on microglia, triggering phagocytic activation and synaptic stripping. In Alzheimer’s disease (AD), amyloid-beta (Aβ) aggregates activate microglia to engulf synapses and neurons. Aβ also induces NLRP3 inflammasome activation, generating ASC specks that seed Aβ plaque formation. Chronic Aβ exposure exhausts microglia, driving their transition into terminally inflammatory microglia (TIM) characterized by impaired clearance and persistent neurotoxicity. Microglial repolarization from pro-inflammatory (M1) to anti-inflammatory (M2) states enhances phagocytosis of neurotoxic debris (e.g., Aβ, α-synuclein) and supports tissue repair via extracellular matrix remodeling. (2) Autoimmune and demyelinating disorders (MS, MSA): Border-associated macrophages (BAMs) amplify neuroinflammation by interacting with microglia and recruiting peripheral mononuclear phagocytes (monocytes, T cells). Prolonged adaptive immunity against autoantigens (e.g., myelin peptides) exacerbates neuronal and oligodendrocyte damage, driving demyelination in multiple sclerosis (MS) and multiple system atrophy (MSA). (3) Therapeutic implications: Targeting brain TRM plasticity (e.g., promoting M2 polarization, blocking TIM formation) or modulating BAM-peripheral immune crosstalk offers strategies to mitigate neurotoxicity and enhance repair.

Microglia in AD exhibit paradoxical roles: on one hand, they exert neuroprotective effects by clearing Aβ plaques, while on the other hand, they may exacerbate pathological damage. Microglia can maintain protective functions through activation of the adhesion G protein-coupled receptor ADGRG1. Specifically, ADGRG1 activates the transcription factor MYC, which upregulates genes related to homeostasis, Aβ phagocytosis (such as C1qa, CD68), and lysosomal degradation (such as CTSB, CTSD), thereby enhancing Aβ clearance and reducing its deposition (Zhu et al., 2025). Moreover, Aβ itself can activate microglia and be phagocytosed, alleviating its accumulation in the brain (Condello et al., 2015); however, Aβ can also trigger apoptosis-associated speck-like protein (ASC) aggregation, promoting plaque formation (Venegas et al., 2017). The TREM2 signaling pathway is crucial for maintaining microglial defense: Aβ binding to TREM2 enhances phagocytosis (Hammond et al., 2019), but prolonged activation leads to TREM2 shedding, weakening Aβ clearance and mediating synapse loss through complement proteins (C1q, C3) (Hong et al., 2016; Shi et al., 2017; Fracassi et al., 2023; Wang et al., 2015; Parhizkar et al., 2019; Pocock et al., 2024). In aged or APOE4-positive brains, microglia in terminal inflammatory states further suppress inflammation resolution, accelerating cognitive decline (Millet et al., 2024) (Figure 2). In addition, AD pathology is not only manifested by Aβ plaque deposition and abnormal tau aggregation, but also by synapse loss. Recent studies have found that neurons can secrete the molecule CX3CL1, which binds to the CX3CR1 receptor on microglia, inducing microglia to release Wnt signals; subsequently, this Wnt activates the Wnt signaling pathway in astrocytes, causing astrocytes to actively retract processes around synapses (Faust et al., 2025). This process, in turn, promotes microglial phagocytosis of synapses. This finding provides new ideas for studying and treating AD and other synaptic dysfunction-related diseases.

Recent single-cell transcriptomic studies have identified a novel microglial subset closely associated with AD pathology—disease-associated microglia (DAM). DAM formation occurs in two steps: first, microglial checkpoint genes are downregulated via a TREM2-independent pathway, and then full activation is driven by TREM2-dependent signaling, endowing microglia with the ability to clear Aβ and limit neurodegeneration. This finding further emphasizes the core role of microglia and TREM2 signaling in AD pathogenesis and progression (Keren-Shaul et al., 2017). Among these, TREM2 is a key innate immune receptor that is highly expressed on microglia in the central nervous system and plays a central role in regulating microglial survival, phagocytic function, and lipid sensing. By associating with the adaptor protein DAP12, TREM2 activates downstream signaling pathways that maintain microglial viability, metabolic homeostasis, and stress resistance under pathological conditions. In addition, TREM2 facilitates the efficient clearance of apoptotic neurons, amyloid-β aggregates, and myelin debris, thereby limiting secondary neuroinflammation and slowing disease progression. Meanwhile, by recognizing diverse lipid ligands and regulating lipid uptake and metabolism, TREM2 enables microglia to adapt to the lipid-rich environments characteristic of neurodegenerative diseases and to acquire disease-associated phenotypes. Collectively, TREM2 serves as a critical regulator of microglial homeostasis and represents a promising therapeutic target for neurodegenerative diseases, although its modulation must be carefully controlled to avoid excessive inflammatory activation (Li et al., 2025). Notably, recent advances in single-cell transcriptomics have further demonstrated that DAM represent a distinct and conserved microglial state associated not only with AD mouse models but also with amyotrophic lateral sclerosis and aging, characterized by downregulation of homeostatic genes and upregulation of lipid metabolism and phagocytosis-related genes, including several human AD risk factors, suggesting a potentially protective role of DAM in regulating disease processes (Cheng and Ho, 2025). Consistent with these findings, a large-scale integrated single-cell and single-nucleus RNA-seq analysis of human brain immune cells across multiple neurodegenerative diseases identified several disease-associated microglial and inflammatory macrophage subpopulations conserved between mouse models and human tissues, highlighting the remarkable plasticity of microglia and their contribution to disease-specific pathological phenotypes (Martins-Ferreira et al., 2025). Importantly, further network-based transcriptomic analyses have revealed substantial heterogeneity within DAM, identifying distinct pro-inflammatory and anti-inflammatory DAM subpopulations with divergent regulatory programs and functional outcomes, indicating that DAM are not a uniform entity but encompass phenotypes that can either exacerbate neuroinflammation or promote amyloid clearance in AD (Rangaraju et al., 2018).

Perivascular macrophages exacerbate A*β*-induced oxidative stress through CD36-dependent ROS generation. Depletion of PVMs or blockade of CD36 reduces cerebrovascular dysfunction (Santisteban et al., 2020; Park et al., 2017; Uekawa et al., 2023). In addition, PVMs drive synapse loss by overactivating microglial phagocytosis through the secretion of secreted phosphoprotein-1 (SPP1) (De Schepper et al., 2023). In Tg2576 mice, bisphosphonate-mediated depletion of PVMs reduced ROS production, thereby reversing Aβ-induced cerebrovascular dysfunction. Bone marrow chimera experiments indicate that PVMs are the primary cell type expressing CD36 and NO2, molecules that significantly contribute to cerebrovascular oxidative stress (Park et al., 2017). PVMs play a key role in upregulating SPP1, whereas perivascular fibroblasts contribute relatively little. In the presence of Aβ oligomers, SPP1 can enhance microglial synapse phagocytosis and increase the expression of phagocytic markers, including complement C1qA chain (C1QA), granulin (GRN), and cathepsin B (CTSB). Notably, knockout of SPP1 prevents synapse loss in AD mouse models (De Schepper et al., 2023). Furthermore, the limited but stable turnover of CD206^+^ BAMs in AD mouse models is noteworthy, as manipulation of these cells may affect AD pathology (Wu et al., 2021). Increasing evidence shows that in AD patients, the significant reduction of LYVE-1 + PVMs is associated with impaired CSF (cerebrospinal fluid) dynamics (Drieu et al., 2022). PVMs maintain efficient CSF waste clearance by phagocytosing perivascular interstitial debris and sustaining high lymphatic flow in the IPAD (intramural periarterial drainage) pathway. Reduced LYVE-1 expression leads to Aβ and other neurotoxic substance accumulation in the brain due to inefficient clearance (Perosa et al., 2022). In addition, tau pathology promotes BAM dysfunction, further causing CSF flow impairment, weakening extracellular tau clearance, exacerbating tau pathology, and ultimately leading to neurodegeneration (Zheng et al., 2025; Drieu et al., 2023) (Figure 2).

### 3.2 Parkinson’s disease

Parkinson’s disease is characterized by the pathological loss or degeneration of dopaminergic neurons in the substantia nigra and the development of neuronal Lewy bodies, associated with risk factors including aging, family history, pesticide exposure, and environmental chemicals (e.g., synthetic heroin use). Its ultimate cause is unknown. PD patients exhibit motor and non-motor symptoms, typically resting tremor, rigidity, bradykinesia, and stooped posture. PD may also involve neurobehavioral disorders (depression, anxiety), cognitive impairment (dementia), and autonomic dysfunction (e.g., orthostatic hypotension, hyperhidrosis) (Beitz, 2014).

In PD, microglia similarly have dual roles. On one hand, M2-type microglia secrete anti-inflammatory cytokines (such as IL-4, TGF-*β*) and neuroprotective factors, aiding neuronal repair, though their efficacy is limited. On the other hand, M1-type microglia release pro-inflammatory cytokines (such as IL-1β, TNF-*α*) and ROS, damaging neurons (Harackiewicz and Grembecka, 2024), and dominate the pathology. Microglia can promote α-synuclein (*α*-Syn) fibril propagation via exosomes, amplifying disease progression (Guo et al., 2020). Oligomeric α-Syn not only recruits microglia via chemotaxis to impair synaptic function and but also triggers an inflammatory cascade of IL-1β/IL-6/TNF-*α* upregulation and excessive ROS production, exacerbating neuronal damage (Ardah et al., 2019; Sarkar et al., 2020). If microglia fail to clear extracellular *α*-Syn promptly, PRRs are further activated, releasing pro-inflammatory mediators and amplifying neuroinflammation. This inflammatory environment promotes α-Syn oligomerization, forming a persistent positive feedback loop, aggravating microglial overactivation and neuronal loss (Allen Reish and Standaert, 2015). Oligomeric *α*-Syn drives microglia toward M1 polarization via TLR2/4, yet their phagocytic function remains essential for α-Syn degradation (Heidari et al., 2022). Beyond mediating α-Syn fibril propagation, recent studies have further demonstrated that microglia-derived extracellular vesicles can transfer cytosolic double-stranded DNA to neurons, thereby activating interferon signaling pathways and inducing neuronal cell death, which highlights an additional pathogenic role of microglial exosomes in neurodegenerative disease progression (Arvanitaki et al., 2024). In addition, increasing evidence indicates that exosomes released from M1 microglia can further exacerbate disease progression by transferring pathogenic molecular cargos, such as regulatory miRNAs, which damage neural tissues, disrupt barrier integrity, and reduce cellular viability (Jiang et al., 2024). Conversely, emerging evidence from Alzheimer’s disease models indicates that anti-inflammatory (M2-like) microglia can release exosomes enriched in neuroprotective miRNAs, such as miR-223, which attenuate neuroinflammation and promote neuronal repair; this process is regulated by RNA-binding proteins such as YB-1 and, together with TREM2-dependent activation of Wnt/β-catenin signaling, highlights the context-dependent, bidirectional roles of microglia-derived exosomes in neurodegenerative disease progression (Wei et al., 2024; Zhu et al., 2025). Overall, microglia-derived exosomes exhibit context-dependent dual roles in neurodegenerative diseases, exacerbating neuronal injury through pro-inflammatory signaling and pathogenic cargos, while under certain conditions also promoting neuroprotection and repair. Notably, the CCL2-CCR2 axis regulates monocyte recruitment, verified in PD models and patients, suggesting a potential therapeutic target to suppress peripheral immune-mediated neurodegeneration (Gao et al., 2023; Gliem et al., 2016) (Figure 2).

In PD models, BAM depletion inhibits *α*-Syn-triggered microglial activation and peripheral immune recruitment, highlighting BAMs’ role in amplifying neuroinflammation (Schonhoff et al., 2023; Frosch et al., 2023). BAMs may act as antigen-presenting cells (APCs) to promote α-Syn-related neuroinflammation, necessary for initiating CD4^+^ T cell responses (Schonhoff et al., 2023). Evidence indicates that immune cell infiltration, recruitment, and antigen presentation in the brain largely depend on BAMs, supporting their potential role in PD pathogenesis (Schonhoff et al., 2023). Furthermore, JAK1/2 inhibitors (such as AZD1480) alleviate α-Syn-induced neuroinflammation by inhibiting the JAK/STAT pathway, demonstrating potential as a PD therapeutic strategy (Qin et al., 2016). Abnormal protein degradation, especially involving α-Syn, may generate immunogenic epitopes during aging and disease progression, triggering immune responses. Specific peptides derived from *α*-Syn act as antigenic epitopes, presented by particular alleles, activating helper and cytotoxic T cells in PD patients (Sulzer et al., 2017). T cells respond not only to extracellular α-Syn natural degradation products in the blood but also to fibrotic *α*-Syn-derived epitopes. This suggests that different forms of α-Syn (natural and fibrotic) and their degradation products may induce distinct T cell-mediated immune responses, promoting PD pathogenesis (Sulzer et al., 2017). Choroid plexus macrophages alleviate inflammation by remodeling epithelial tight junctions, whereas meningeal macrophages clear *α*-Syn via lymphatic-like drainage (Xu et al., 2024; Zou et al., 2019; Bogale et al., 2021) (Figure 2).

### 3.3 Huntington’s disease

Huntington’s disease (HD) pathogenesis originates from CAG repeat expansion in the huntingtin protein (HTT), and mutant HTT-driven microglial inflammation exacerbates neurodegeneration (The Huntington's Disease Collaborative Research Group, 1993; Sharp et al., 1995; Saba et al., 2022). Increasing evidence indicates that microglia play an active and critical role in the onset and progression of HD (Isop et al., 2023; Nayak et al., 2011). Analyses of patient samples have shown that microglial activation can be detected prior to the onset of clinical symptoms, suggesting that microglia may be involved in the early pathological processes of the disease (Chang et al., 2015). HTT is not only expressed in neurons but also induces cell-autonomous dysfunction in microglia, leading to excessive activation and the release of pro-inflammatory cytokines, such as IL-1*β*, IL-6, and TNF-α, thereby exacerbating neuroinflammation and neuronal damage (Valekova et al., 2016). In addition, microglia can respond to mHTT-induced neuronal pathology through non-cell-autonomous mechanisms, further amplifying neurotoxic effects. In HD, microglia predominantly exhibit a pro-inflammatory M1 phenotype, whereas the reparative and phagocytic M2 phenotype is relatively insufficient; this imbalance is considered to be closely associated with disease progression (Hsiao et al., 2014). Moreover, multiple signaling pathways, including NF-κB, the kynurenine pathway, and endocannabinoid receptor signaling, are involved in regulating microglial activation and have been implicated in HD-related neurodegenerative pathology (Träger et al., 2014; Guillemin et al., 2005; Chiarlone et al., 2014). Collectively, microglia may exacerbate HD pathology through inflammatory amplification while exerting protective effects under specific conditions, and their activation state and functional modulation are therefore regarded as important potential therapeutic targets for HD. Therefore, inhibiting microglial activation helps maintain synaptic integrity and delays symptom onset. Additionally, HD disrupts neuron–microglia communication via the CX3CL1-CX3CR1 axis: CX3CL1 levels decrease in the human striatum and R6/1 mouse striatum, accompanied by abnormal PSD-95 phagocytosis (Pawelec et al., 2020; Kim et al., 2020; Abedrabbo et al., 2025). Meanwhile, upregulation of kynurenine-3-monooxygenase (KMO) in microglia promotes L-kynurenine conversion to the neurotoxic metabolite 3-hydroxykynurenine, indicating the kynurenine pathway as a potential therapeutic target (Lu et al., 2020).

### 3.4 Multiple sclerosis

MS is an autoimmune-mediated neurodegenerative disease characterized by inflammatory demyelination (Wang et al., 2023). It is mediated by activated T cells, with growing evidence showing significant contributions from B cells and innate immune cells. This disease is thought to result from complex interactions between genetic and environmental factors (Yamout and Alroughani, 2018).

In early and progressive stages of MS, microglia exhibit a pro-inflammatory phenotype, releasing IL-6, IL-1β, IL-1α, IL-12, and TNF-α, exacerbating neuroinflammation (Zhang et al., 2021; Li et al., 2022); in later stages, microglia shift to an anti-inflammatory state, secreting IL-10, IL-13, and TGF-β, promoting neuroprotection (Nan et al., 2024). Inflammatory mediators expressed by microglia, such as LRP1, P2X7R, and TLR4, drive disease progression in early stages. LRP1 deletion promotes TNF-α release, enhancing inflammation (Chuang et al., 2016). P2X7R overexpression is a hallmark of MS pathology, driving cytokine storms (IL-1β, IL-6, TNF-α) and sustaining neuroinflammatory damage (Sidoryk-Węgrzynowicz and StruŻyńska, 2021; Grygorowicz and StruŻyńska, 2019). LPS induces TLR4 overexpression in microglia, promoting their pro-inflammatory polarization and accelerating disease progression (Zusso et al., 2019). In neurodegenerative diseases, miRNA dysregulation often skews microglia toward M1 or neurotoxic phenotypes. As non-coding RNAs, miRNAs regulate microglial activation status, influencing neuroinflammatory processes. miR-124 exerts neuroprotective effects and inhibits M1 microglial activation; conversely, miR-155 is upregulated by inflammatory stimuli, further activating more M1 microglia and exacerbating inflammation. Multiple studies show that during MS progression, the anti-inflammatory marker miR-124 is significantly downregulated, while the pro-inflammatory marker miR-155 is markedly upregulated (Asl et al., 2025). Therefore, therapeutic strategies that regulate miRNAs to shift more microglia toward the M2 phenotype may offer new approaches for MS treatment. In late-stage MS, microglia-specific expression of chimeric galectin-3 (Gal-3) mediates M2 phenotype switching and TREM-2b-dependent myelin phagocytosis, alleviating neuroinflammation (Thomas and Pasquini, 2018). Additionally, microglial secretion of IFN-β can synergistically enhance phagocytic activity and related gene expression, providing a dual strategy for MS therapy (Kocur et al., 2015) (Figure 2). Foamy macrophages are macrophages that acquire a characteristic foamy appearance due to the accumulation of abundant cytoplasmic lipid droplets following the ingestion of large amounts of lipid-rich material, particularly myelin components. In the early active lesions of central nervous system inflammatory and demyelinating diseases, such as multiple sclerosis, these cells are abundantly present and are primarily derived from infiltrating macrophages and resident microglia (Teuber-Hanselmann et al., 2020).

In MS, PVMs near lesion sites express antigen-presenting molecules and heat shock proteins (HSPs), promoting T cell activation and lesion progression (Ostkamp et al., 2022; Miedema et al., 2022; Schläger et al., 2016; Kim et al., 2024). In addition, BAMs promote MS pathology by upregulating interleukin-9 (IL-9). Autopsy studies show elevated IL-9 levels in the CSF of MS patients, and flow cytometry confirms high IL-9 expression in brain-associated macrophages (Donninelli et al., 2020). Therapeutic interventions targeting BAM dysfunction show potential. For example, treatment with 4-phenylbutyric acid (4-PBA) can prevent pathological peroxisome damage in BAMs, thereby reducing demyelination and axonal loss, representing a potential strategy to halt MS progression (Roczkowsky et al., 2022). Furthermore, foamy macrophages accumulate in MS-affected brain regions, and targeting their lipid phagocytosis may promote myelin regeneration, as certain BAM subtypes appear to be involved in this process (Grajchen et al., 2018; Haidar et al., 2022) (Figure 2).

### 3.5 Ischemic stroke and other brain injuries

Ischemic stroke (IS) is a detrimental neurological disorder, with the initial damage being cerebral infarction. Under insufficient blood supply, reversible tissue functional loss occurs, and if prolonged, infarction results in loss of neurons and supportive structures. Ischemia triggers a cascade of events, starting with electrical dysfunction, followed by membrane dysfunction, calcium influx leading to calcium-dependent excitotoxicity, ROS generation, and ultimately cell membrane disruption and lysis (Feske, 2021). Traumatic brain injury (TBI) is an acquired brain injury caused by external mechanical force, potentially resulting in temporary or permanent damage (Capizzi et al., 2020). It has high neurological morbidity, with severe TBI associated with high mortality and poor neurological outcomes (Robinson, 2021). Additionally, it is the most common cause of neuro- and neurosurgical death and survivor disability in children and young adults (Figaji, 2023). Radiation-induced brain injury (RIBI) is a neurological disorder caused by radiotherapy for malignant tumors, with incompletely understood pathogenesis (Wang et al., 2024). It is the most severe complication of head and neck radiotherapy, significantly affecting quality of life. Currently, there is no effective treatment for RIBI patients (Zhang et al., 2022).

Within 3 h of IS, microglia rapidly change morphology (enlarged soma, reduced dendritic number and complexity), transitioning from a “surveillance” to an “activated” state, secreting large amounts of inflammatory factors. They are the main source of inflammatory mediators during the hyper-acute phase of stroke (within 3 h), directly driving early neuroinflammation (Bourne et al., 2025), suggesting that future therapies could focus on microglia in the ultra-acute phase of stroke. Local post-stroke inflammation induces microglial activation, characterized by upregulation of surface markers (MHC II, CD68, CD45) (Ransohoff and Perry, 2009; Heindl et al., 2018). The duration of ischemia determines microglial dynamics: brief ischemia leads to reversible shortening of processes, whereas severe ischemia induces irreversible amoeboid transformation (Ju et al., 2018; Li et al., 2013). Recent studies indicate that depending on their polarization state, microglia after stroke can both exacerbate neuroinflammation and promote angiogenesis and neurogenesis (Qin et al., 2019; Hardiman et al., 2017) (Figure 2). BAMs also promote key pathophysiological changes in IS, such as granulocyte recruitment, increased VEGF expression, and elevated permeability of leptomeningeal and cortical vessels (Pedragosa et al., 2018). After induced stroke, CD163^+^ BAMs expand and migrate to ischemic parenchyma, exhibiting a pro-inflammatory phenotype. Simultaneously, CD169^+^ PVMs also expand during stroke but are eventually replaced by bone marrow-derived infiltrating cells. This finding is confirmed in human studies, where accumulation of CD163^+^ macrophages is similarly observed in ischemic regions (Rajan et al., 2020). Post-stroke, CD163^+^ BAMs acquire pro-inflammatory functions and drive leukocyte infiltration and BBB leakage through VEGF upregulation (Sun and Jiang, 2024) (Figure 2).

In TBI, primary injury directly causes local tissue and cellular damage (Simon et al., 2017). Damaged neurons release damage-associated molecular patterns (DAMPs), triggering microglia to secrete cytokines and chemokines (Disabato et al., 2016; Lafrenaye et al., 2015; Alam et al., 2020), inducing neuroinflammation (Wofford et al., 2019). Activated microglia release TNF-*α*, IL-6, and IL-1β, participating in early neuroinflammatory responses (Simon et al., 2017; Kaelber et al., 2016). The injury cascade begins with ROS-mediated axonal degeneration, further activating microglia and maintaining inflammation through TNF-α/IL-6/IL-1β signaling (Simon et al., 2017; Witcher et al., 2015). Breaking this positive feedback loop can alleviate secondary injury. RIBI causes significant microglial structural remodeling, including soma enlargement, process shortening, and enhanced phagocytic activity (Wang et al., 2017; Sheu et al., 2023; Monsorno et al., 2022). Radiotherapy induces microglial pro-inflammatory polarization, manifested by increased TNF-α-positive cells, reduced dendritic spine density, and impaired synaptic plasticity (Wang et al., 2024; Wilke et al., 2018). Inflammatory factors such as TNF-α, IL-16, MIP-1α, and MMP-9 further compromise BBB integrity, allowing peripheral immune cell infiltration (Wang et al., 2024). Additionally, NADPH oxidase (NOX) is a key regulator of immune response and microglial oxidative stress (Simpson and Oliver, 2020). Microglia produce neurotoxic ROS via NOX, amplifying the production of multiple neurotoxic and pro-inflammatory cytokines (Chen et al., 2021). Microglia mainly express NOX2, and also express NOX4 (Cooney et al., 2013; Ma et al., 2018; Xie et al., 2020). After brain injury, NOX2-mediated ROS induce ferroptosis and inflammation via NF-κB activation (Hegdekar et al., 2023; Wang et al., 2017); NOX4 activation is also associated with oxidative stress and neuroinflammation (Ma et al., 2017; Yauger et al., 2019; Bano et al., 2025).

## 4 Therapeutic targeting of BRMs in neurological diseases

BRMs, including microglia and BAMs, play key roles in maintaining CNS homeostasis and neuroimmune regulation. In recent years, therapeutic strategies targeting BRM functional regulation have gradually emerged as important approaches to alleviate neuroinflammation and neurodegenerative pathology. Based on mechanisms of action and intervention pathways, these strategies can be divided into the following major categories.

### 4.1 Inflammasome and receptor-modulating drugs

#### 4.1.1 NLRP3 inflammasome inhibitors

Excessive activation of the NLRP3 inflammasome is considered a critical driver of pro-inflammatory microglial responses in AD. The synthetic molecule N, N′-diacetyl-p-phenylenediamine (DAPPD) effectively inhibits NLRP3 activity, enhances microglial phagocytic function, and reduces Aβ deposition (Park et al., 2019). The selective NLRP3 inhibitor Dapansutrile (OLT1177) has been shown to alleviate microglial overactivation in AD models, improve synaptic plasticity, reduce Aβ plaque burden, and mitigate post-hemorrhagic cerebral edema (Lonnemann et al., 2020; Fang et al., 2024). In ischemic stroke (IS), NLRP3 inhibition similarly promotes M2-type microglial polarization and protects neurons (Sapkota and Choi, 2022; Wang et al., 2022), with neuroprotective effects partially dependent on anti-inflammatory responses mediated by Indoleamine 2,3-dioxygenase 1 (IDO-1) upregulation (Ji et al., 2021). Moreover, recent studies indicate that NLRP3 inflammasome inhibition is not only effective in neurodegenerative diseases but also improves cognitive deficits associated with depression. In a chronic unpredictable mild stress (CUMS)-induced depression mouse model, the NLRP3-specific inhibitor MCC950 (10 mg/kg, i.p.) significantly alleviated depressive-like behaviors and cognitive impairment, restoring hippocampal neuronal Aβ metabolism pathways and abnormal tau phosphorylation. *In vitro* experiments also confirmed that inhibiting microglial NLRP3 activity reduces IL-1β release, decreases neuronal damage, and restores synaptic function. These findings suggest that NLRP3-mediated inflammatory responses may be a shared neuroimmune mechanism in depression and AD, and NLRP3 inhibitors (such as MCC950) hold potential as common therapeutic targets for multiple neurological diseases (Li et al., 2023).

#### 4.1.2 TREM2 signaling agonists

TREM2 is a core receptor regulating microglial activation and Aβ clearance. Activating TREM2 can reprogram microglia into a protective phenotype, enhance Aβ phagocytosis, reduce neuronal loss, and improve cognitive deficits (Wang et al., 2022; Xu et al., 2019). Furthermore, the STING pathway can upregulate TREM2 expression via cGAMP stimulation, promoting M2 polarization and enhancing neuroprotection (Xu et al., 2019). The cGAS–STING pathway is a central innate immune signaling axis responsible for sensing cytosolic DNA and plays an important role in neuroinflammation and neurodegenerative diseases (Li et al., 2025). Upon recognition of aberrant DNA in the cytoplasm, cyclic GMP–AMP synthase (cGAS) catalyzes the production of the second messenger cGAMP, which subsequently activates the endoplasmic reticulum–resident adaptor protein STING. Activated STING undergoes conformational changes and translocates to the Golgi apparatus, where it recruits and activates TANK-binding kinase 1 (TBK1), leading to phosphorylation and nuclear translocation of interferon regulatory factor 3 (IRF3) and ultimately inducing the transcription of type I interferons and multiple pro-inflammatory cytokines (Wang et al., 2024). In the central nervous system, components of the cGAS–STING pathway are widely expressed in microglia, astrocytes, and neurons, and their activation is highly dependent on cell type and pathological context (Zhang et al., 2024). In early AD, combined use of TREM2 agonists and Aβ antibodies significantly enhances plaque clearance, suppresses microglial “overactivation,” and prevents synaptic damage (De Weerd et al., 2025). Recent clinical studies targeting TREM2 provide direct evidence for its therapeutic potential. AL002 is a humanized IgG1 monoclonal antibody specifically targeting TREM2. In preclinical studies in non-human primates, weekly intravenous injection of AL002 for 4 weeks dose-dependently reduced soluble TREM2 levels in CSF and increased biomarkers of TREM2 signaling. In the Phase 1 INVOKE-1 (NCT03635047) clinical trial in healthy volunteers, a single intravenous injection of AL002 also dose-dependently decreased CSF sTREM2 while enhancing markers of TREM2 signaling and microglial recruitment, demonstrating CNS penetration and good safety and tolerability (Long et al., 2024). These results indicate that AL002 effectively acts on TREM2 in humans and supports its continued development in AD and other neurodegenerative diseases. However, due to limited BBB penetration, TREM2-targeted therapy still faces challenges of high dosing and low delivery efficiency (Lin et al., 2024; Kariolis et al., 2020). To address this, researchers have developed an Antibody Transport Vehicle (ATV) targeting transferrin receptor (TfR)-mediated BBB transport, incorporating asymmetric Fc mutations (ATVcisLALA) to avoid anemia and vascular injury, thereby improving safety and efficacy (Pizzo et al., 2025).

### 4.2 Metabolism and energy-regulating drugs

Energy metabolism pathways play an important role in regulating microglial immune status. Studies show that in brain tissue with enhanced Aβ clearance, both oxidative phosphorylation and glycolysis in microglia are reduced, suggesting that metabolic intervention can alleviate chronic neuroinflammation (Van Olst et al., 2025). Clinical studies have found that plasma AMPKα1 levels are significantly decreased in patients with mild cognitive impairment (MCI) and AD, while AMPKα2 levels remain unchanged, suggesting a key role of AMPKα1 in early AD energy metabolism regulation (Wang et al., 2020). Plasma AMPKα1 levels significantly correlate with neuroimaging biomarkers (e.g., AD-characteristic cortical thickness), and ROC analysis indicates AMPK*α*1 effectively distinguishes MCI/AD patients from healthy controls, highlighting its potential clinical diagnostic and predictive value (Wang et al., 2020). These clinical findings further support strategies to activate AMPKα1 to improve energy metabolism and microglial function, thereby delaying AD pathology. The AMPKα1 activator DW14006 promotes M2 polarization and enhances Aβ phagocytosis (Lv et al., 2020). Additionally, IDO-1 is a key rate-limiting enzyme of the tryptophan–kynurenine pathway and plays a critical role in regulating immune responses, cellular metabolism, and brain immune homeostasis under inflammatory conditions by catalyzing tryptophan degradation (Chang and Yang, 2019). Activation of IDO-1 not only suppresses effector immune cell functions through tryptophan depletion but also induces an immunosuppressive state via the kynurenine–aryl hydrocarbon receptor (KYN–AhR) signaling axis. In the central nervous system, IDO-1 and its metabolites contribute to the regulation of neuroinflammatory responses and are closely associated with the pathogenesis of neurodegenerative diseases by affecting neurotransmitter balance, excitotoxicity, and glial cell function (Mei et al., 2020). In the central nervous system, IDO-1 and its metabolites contribute to the regulation of neuroinflammatory responses and are closely associated with the pathogenesis of neurodegenerative diseases by affecting neurotransmitter balance, excitotoxicity, and glial cell function (Salminen, 2022). Recent studies show that IDO-1 is primarily distributed in meningeal and perivascular macrophages/microglia, rather than in the brain parenchyma, in human brain, APP/PS1 mice, and glioblastoma tissues. Functional experiments indicate that inhibiting IDO-1 (e.g., using 1-MT or INCB24360) significantly reduces immune cell pinocytosis and phagocytosis, promotes IL-1β secretion, and inhibits NLRP3 expression, weakening anti-inflammatory and clearance functions (Ji et al., 2021; Park et al., 2020); conversely, sustained IDO-1 expression enhances PVM phagocytic activity and prevents excessive inflammatory activation (Ji et al., 2021). This suggests that IDO-1 may play a key defensive role in BBB protection and A*β* clearance by maintaining local immune-metabolic balance and phagocytic capacity.

### 4.3 Anti-inflammatory and receptor-targeting small molecules

Small molecules demonstrate high feasibility and safety in regulating microglial activation. Anti-inflammatory drugs such as the 5-HT₂A antagonist desloratadine and microRNA-146a can promote M2 polarization and enhance Aβ clearance (Liang et al., 2021; Lu et al., 2021). Recent studies further confirm that desloratadine, in addition to being a selective 5-HT₂A receptor antagonist, effectively ameliorates AD pathology in APP/PS1 mice. Its mechanism includes promoting microglial phagocytosis and degradation of Aβ, inhibiting pro-inflammatory responses, and inducing anti-inflammatory M2 polarization. Desloratadine also activates the 5-HT₂A R/cAMP/PKA/CREB/Sirt1 signaling pathway, stimulating autophagy, inhibiting neuroinflammation, and promoting glucocorticoid receptor (GR) nuclear translocation, thereby upregulating transcription of phagocytic receptors TLR2 and TLR4, further enhancing microglial phagocytic activity (Lu et al., 2021). In PD, vitamin D has been found to inhibit excessive microglial activation and promote M2 anti-inflammatory polarization, protecting dopaminergic neurons from inflammatory and oxidative damage (Calvello et al., 2017). Importantly, a recent randomized, double-blind, placebo-controlled clinical trial (ClinicalTrials.gov: NCT06539260) demonstrated that vitamin D₃ supplementation significantly improved immune and motor function in PD patients: after 3 months, serum 25(OH)D₃ levels increased, Th17 cell proportions decreased, Treg cell proportions increased, indicating restored immune balance; simultaneously, UPDRS and UPDRS-III scores decreased significantly, alleviating motor symptoms. This study provides clinical evidence that vitamin D₃ may improve PD progression by modulating Th17/Treg balance, supporting its neuroprotective and immunoregulatory roles (Li et al., 2025).

Myeloperoxidase (MPO) inhibitors also show clinical potential in controlling microglia-associated oxidative stress and chronic inflammation. MPO, secreted by activated microglia, generates ROS whose sustained activation leads to neuronal damage and disease progression. A recent Phase IIa randomized, placebo-controlled multi-center study demonstrated that the selective irreversible MPO inhibitor AZD3241 significantly reduced [^11C]-PBR28 binding in the substantia nigra-striatum region of PD patients (13–16% decrease, *p* < 0.05), indicating reduced microglial activation. After 8 weeks of treatment, the drug was safe and well tolerated, further supporting MPO inhibition as a means to alleviate neuroinflammation and potentially modify disease, providing key clinical evidence for microglial-targeted therapy in neurodegenerative diseases (Murphy and Lynch, 2023).

Additionally, CB₂ receptor agonists (e.g., SR144528) selectively inhibit striatal microglial overactivation, improving motor function and neuroinflammation (Gao et al., 2023). Recent reviews indicate that the CB₂ receptor, a key component of the endocannabinoid system, can modulate neuroinflammatory responses in PD. Extensive preclinical evidence shows that CB₂ agonists exert neuroprotective effects in various PD animal models by inhibiting microglial activation and reducing pro-inflammatory cytokine release. Altered CB₂ expression has also been observed in PD patient brain tissues, suggesting its potential as a biomarker for neuroinflammatory progression. However, direct clinical validation is still lacking, and further systematic clinical studies are needed to clarify the precise role of CB₂ in PD pathogenesis and intervention (Basile and Mazzon, 2022). In summary, small molecules targeting microglia-related signaling pathways (such as 5-HT₂A, MPO, and CB₂ receptors) exhibit multifaceted regulatory effects in neurodegenerative diseases, including inflammation inhibition, neuroprotection, and improvement of cognitive and motor function. Combining clinical validation with molecular mechanism studies in the future may provide new avenues and strategies for disease-modifying therapies of neuroinflammation-mediated disorders.

### 4.4 miRNA and gene regulation strategies

MiRNA-based therapies have shown significant immunomodulatory potential in neurodegenerative diseases. In a MS model, non-viral vector-mediated silencing of miR-155 can upregulate its target SOCS-1, reduce microglial secretion of NO, TNF-*α*, and IL-6, and significantly decrease local inflammation levels (Cardoso et al., 2012). Moreover, downregulation of miR-124 in spinal microglia is closely associated with enhanced M1 phenotype and increased pro-inflammatory factors. Intrathecal injection of miR-124 mimics can reverse this inflammatory phenotype, inhibit neuropathic pain, and promote neural repair (Willemen et al., 2012). Although this strategy is highly specific, it is still limited by low miRNA delivery efficiency and individual variability (Asl et al., 2025). MiRNA-based therapies have shown significant immunomodulatory potential in neurodegenerative diseases. In MS models, non-viral vector-mediated silencing of miR-155 can upregulate its target SOCS-1, reduce microglial secretion of NO, TNF-α, and IL-6, and significantly decrease local inflammation levels (Cardoso et al., 2012). This finding is consistent with *in vitro* results: in N9 microglial cells, LPS stimulation strongly upregulates miR-155 expression, leading to downregulation of SOCS-1 protein and increased production of NO and inflammatory cytokines; conversely, using antisense oligonucleotides to inhibit miR-155 restores SOCS-1 expression and significantly reduces the release of inflammatory mediators, thereby decreasing microglia-mediated neuronal death, suggesting that miR-155 has a pro-inflammatory role in microglial activation and its inhibition has potential neuroprotective effects (Cardoso et al., 2012).

Furthermore, downregulation of miR-124 in spinal microglia is closely associated with enhanced M1 phenotype and increased pro-inflammatory factors. Intrathecal injection of miR-124 mimics can reverse this inflammatory phenotype, inhibit neuropathic pain, and promote neural repair (Willemen et al., 2012). Despite the high specificity of this strategy, it is still limited by low miRNA delivery efficiency and individual variability (Asl et al., 2025). Notably, clinical studies have also validated the therapeutic potential of miRNA regulation in MS patients. Sievers et al. analyzed the miRNA profiles of peripheral blood mononuclear cells from 74 patients with relapsing–remitting MS (RRMS) and 32 healthy controls and found abnormal expression of immune-related miRNAs such as miR-326, miR-155, miR-146a, and miR-142-3p. Further comparison showed that interferon-*β* treatment did not significantly alter these miRNA levels, whereas patients treated with glatiramer acetate (GA) showed a significant decrease in miR-146a and miR-142-3p expression, suggesting that GA can partially restore dysregulated miRNA expression and thus reestablish immune homeostasis (Waschbisch et al., 2011). These results provide key evidence for the feasibility of miRNA regulation in clinical therapy: miRNAs are not only involved in the pathogenesis of MS but may also serve as important targets for disease repair and immune intervention. Combined animal and clinical study results indicate that miRNA-targeted therapy has translational potential from experimental models to clinical applications in regulating neuroinflammation and neuroprotection.

### 4.5 Cell and exosome therapies

#### 4.5.1 Autologous cell transplantation therapy

Injection of autologous bone marrow mononuclear cells (BM-MNCs) within 72 h after TBI can induce apoptosis of pro-inflammatory microglia and improve cognitive function (Scott et al., 2021). In rodent TBI models, transplantation of pluripotent adult progenitor cells can drive M2 polarization through IL-4/IL-10 signaling, reducing secondary immune damage (Goldman et al., 2022). Notably, clinical studies further confirm the feasibility and safety of this strategy. In a randomized, double-blind, placebo-controlled Bayesian dose-escalation clinical trial (NCT01851083), researchers administered autologous BM-MNC infusion to children with severe TBI within 48 h of injury. The results showed that the BM-MNC treatment group had significantly shorter intensive care unit stays, mechanical ventilation duration, and intracranial pressure monitoring time; MRI assessment indicated that white matter volume in the treatment group was significantly better than in the control group, and callosal fiber connections were partially preserved (Cox et al., 2024). These results suggest that early autologous BM-MNC reinfusion is not only safe and feasible but may also improve long-term neurological outcomes by reducing neuroinflammation and protecting white matter structures and the blood–brain barrier.

In ischemic and hemorrhagic stroke, a clinical trial isolated autologous peripheral blood mononuclear cells (PBMCs) from non-acute stroke patients, polarized them toward the M2 phenotype in vitro, and administered them intrathecally following dexamethasone pretreatment. This approach resulted in a significant improvement in NIHSS scores in 75% of treated patients compared with only 18% in the control group, with no adverse events related to cell therapy reported (Chernykh et al., 2016). Consistent with these clinical findings, adoptive transfer of bone marrow–derived macrophages polarized toward the M2 phenotype with IL-4 and M-CSF in vitro significantly alleviated symptoms of experimental autoimmune encephalomyelitis (EAE) in C57BL/6 mice (Chu et al., 2021). These studies indicate that ex vivo polarization and autologous reinfusion of macrophages represent a feasible and effective translational strategy for neurological disorders, with therapeutic efficacy validated across different species and disease models. However, M1 and M2 macrophages exhibit distinct marker profiles in mice (M1: iNOS/CD40; M2: CD206/Arg-1), while in humans, additional markers are preferentially expressed (M1: iNOS/CD40/NOX1; M2: CD206/CD163), and co-expression suggests intermediate phenotypes, warranting further human-specific validation (Jäckle et al., 2020). Taken together, autologous reinfusion and immune-reprogramming therapies based on peripheral monocytes/macrophages hold promise as safe and translatable therapeutic strategies for neurological diseases such as TBI, stroke, and autoimmune encephalopathies, by promoting anti-inflammatory M2 polarization, suppressing excessive microglial activation, and repairing the blood–brain barrier.

#### 4.5.2 Peripheral mononuclear phagocyte–mediated brain repair

The choroid plexus regulates the infiltration of peripheral monocyte-derived macrophages (MDMs) into the brain, where they differentiate into macrophages that cooperate with (BAMs to repair vascular damage and restore BBB integrity; Xu et al., 2024; Kim and Luster, 2015; Wang and Kubes, 2016; Brazil et al., 2019; Choi et al., 2023). In AD models, intravenous administration of monocytes from young mice enhances Aβ clearance and improves cognitive performance (Hohsfield and Humpel, 2015). Moreover, inhibition of TGF-β/Smad2/3 signaling facilitates macrophage recruitment to Aβ plaques, accelerating the phagocytic removal of pathological proteins (Weitz and Town, 2012). These infiltrating macrophages exhibit pro- or anti-inflammatory phenotypes depending on the local microenvironment and their interaction with microglia, thereby dynamically modulating cerebral immune responses (Passaro et al., 2021). Engineered monocytes overexpressing angiotensin-converting enzyme (ACE) further reduce neurotoxic Aβ42–43 levels, limit amyloid plaque formation, suppress astrocyte proliferation, and consequently improve cognitive outcomes in AD mice (Zuroff et al., 2017; Liu et al., 2019; Koronyo-Hamaoui et al., 2020). However, excessive infiltration of phagocytes may exacerbate demyelinating pathology in MS (Lassmann, 2018). Although short-term cognitive benefits have been reported, age-dependent variations in phagocytic activity remain controversial, requiring further investigation into long-term mechanisms and age-adapted therapeutic strategies (Hohsfield and Humpel, 2015).

#### 4.5.3 Chimeric antigen receptor macrophage (CAR-M) therapy

Chimeric antigen receptors (CARs) are synthetic receptors capable of recognizing target antigens independently of MHC presentation, enabling cytotoxic immune cells to be redirected toward specific pathological antigens (Pan et al., 2022). In AD models, intrahippocampal administration of anti-Aβ CAR macrophages (CAR-Ms) engineered to secrete M-CSF resulted in prolonged survival and markedly enhanced Aβ clearance (Kim et al., 2024). These findings highlight the therapeutic potential of CAR-Ms in neurodegenerative disorders. However, significant challenges remain for their application in the CNS: the hostile neural microenvironment restricts CAR-M viability, and activation can trigger excessive pro-inflammatory cytokine responses (Spiteri et al., 2022; Li et al., 2024). Therefore, future strategies should focus on controlled activation, signal modulation, and microenvironment-adaptive engineering to optimize CAR-M safety and durability.

#### 4.5.4 Nanoparticle- and macrophage membrane–coated drug delivery systems

Nanoparticles (NPs) and exosomes provide innovative platforms for brain-targeted drug delivery. Macrophage-loaded NPs exhibit improved lesion-homing capability but face intracellular degradation of payloads; improved designs include “backpack”-type NPs attached to the cell surface or NPs camouflaged with macrophage membranes for controlled drug release (Ejigah et al., 2022; Wang et al., 2023; He et al., 2021). Furthermore, NPs cloaked with macrophage membranes retain biocompatibility, immune evasion, and receptor-mediated chemotaxis and targeting properties, thereby achieving efficient BBB penetration and targeted delivery (Xuan et al., 2015; Cao et al., 2016; Rao et al., 2017). This approach has demonstrated significant pharmacokinetic advantages and therapeutic potential in models of brain tumors, AD, and traumatic brain injury.

#### 4.5.5 MSC exosome delivery system

Mesenchymal stem cell (MSC)-derived exosomes, as miRNA delivery vehicles, can transfer anti-inflammatory miRNAs (such as miR-216a-5p, miR-125a, miR-146a-5p, and miR-223-3p) to inhibit microglial activation and related neural damage (Liu et al., 2020; Chang et al., 2021; Zhang et al., 2021; Zhao et al., 2020). Adipose-derived MSC exosomes can also induce microglial secretion of TGFβ and promote M2 polarization, alleviating brain edema and neuronal injury (Xu et al., 2020). Furthermore, recent reviews indicate that MSC-derived exosomes (MSC-Exos) exhibit significant immunomodulatory and neuroprotective effects in acute CNS injuries, including stroke and TBI. MSC-Exos can regulate pro-inflammatory microglial phenotype balance, suppress persistent neuroinflammation, maintain blood–brain barrier integrity, and promote neural repair (Liu et al., 2023). The review further emphasizes that MSC-Exos have demonstrated neuroprotective and reparative potential in animal models and are clinically feasible due to their safe origin, low immunogenicity, and suitability as a cell-free therapy for early CNS injury functional recovery interventions (Liu et al., 2023). In summary, MSC-Exos, by delivering miRNAs and modulating microglial activation, show broad prospects as biologic response modifier (BRM) targeted therapies at both basic and clinical levels.

**Figure 3:**
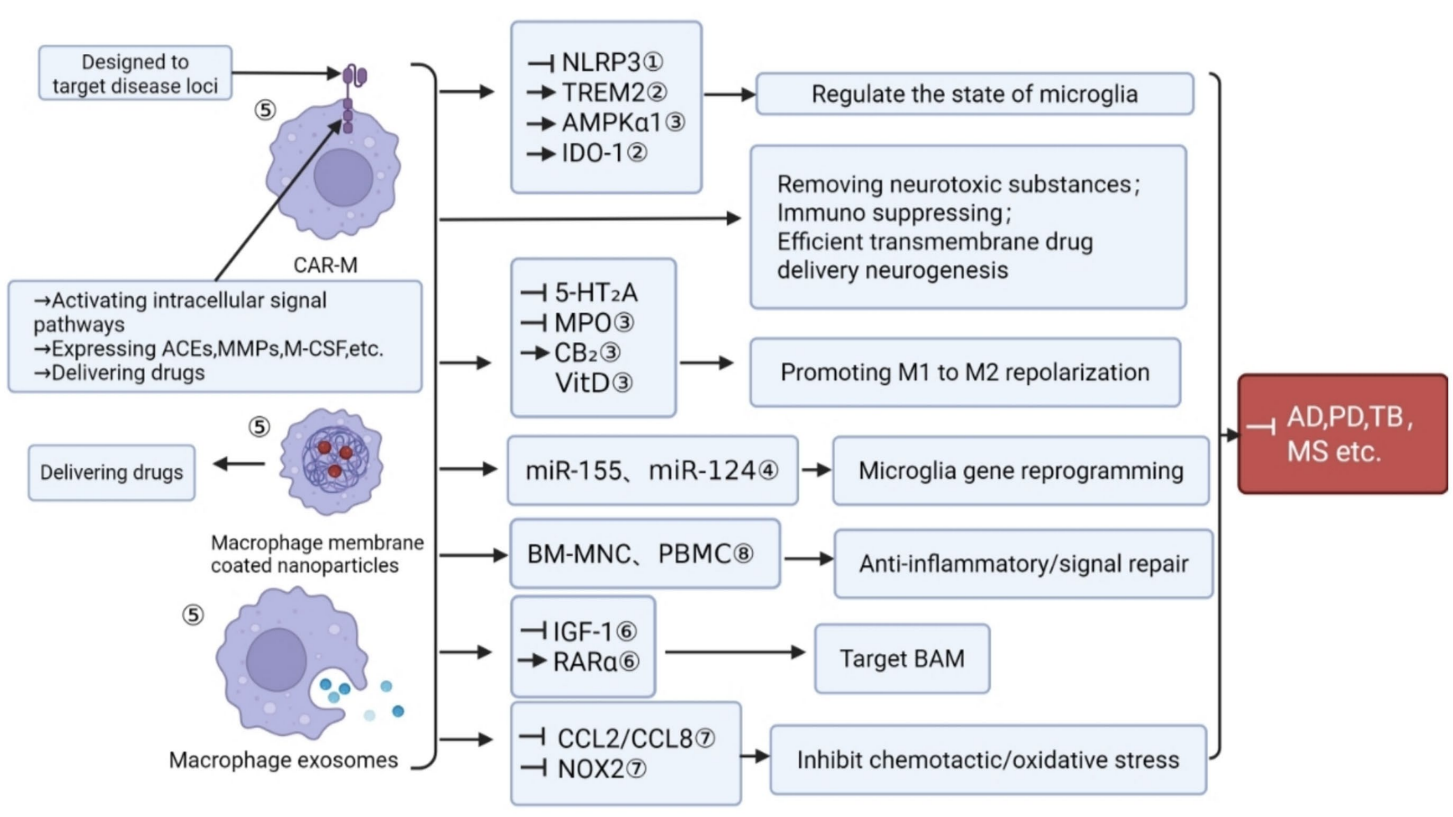
Overview of multidimensional therapeutic strategies targeting BRMs. Precise modulation of BRM function has emerged as a promising therapeutic approach for neurodegenerative diseases. The principal strategies include: ① NLRP3 inflammasome inhibitors reduce microglial overactivation, promote M2 polarization, and alleviate Aβ deposition and neuroinflammation in AD, stroke, and depression models. ② TREM2 signaling agonists (e.g., AL002) enhance microglial phagocytosis, promote neuroprotection, and improve cognitive function; novel antibody transport vehicles (ATVs) enhance BBB penetration and therapeutic efficacy. ③ Metabolic and receptor-targeted small molecules (such as AMPKα1 activators, vitamin D_3_, desloratadine, MPO inhibitors, and CB_2_ receptor agonists) regulate microglial energy metabolism, polarization, and neuroinflammation, improving motor and cognitive outcomes. ④ MiRNA and gene regulation strategies (e.g., miR-155 inhibition, miR-124 overexpression) modulate microglial inflammatory phenotypes, restore immune homeostasis, and demonstrate translational potential from animal models to clinical applications. ⑤ Cell and exosome therapies utilize autologous mononuclear cells, engineered macrophages, CAR-macrophages, and MSC-derived exosomes to promote M2 polarization, repair the BBB, and suppress neuroinflammation. ⑥ Vascular-associated strategies targeting BAMs regulate vascular integrity and inflammatory responses via IGF-1 and RARα signaling, promoting remyelination and neuroprotection in chronic MS models. ⑦ Chemokine and oxidase inhibitors (e.g., CCL2/CCL8 blockade, NOX2 inhibitor NCATS-SM7270) suppress neuroinflammatory signaling and reduce cortical neuronal damage, offering selective neuroprotection. ⑧ Antibody and B cell-targeted therapies (e.g., ocrelizumab, ofatumumab, siponimod) reduce CNS infiltration of B cells, inhibit microglial–lymphocyte interactions, and alleviate chronic inflammation in MS and related neurodegenerative disorders.

Peripherally derived exosomes engineered ex vivo can also be utilized to alleviate neuroinflammation. Macrophage-derived exosomes (M1-Exos and M2-Exos) are capable of reprogramming immune responses: M1-Exos can repolarize M2 macrophages toward a pro-inflammatory phenotype via the caspase-3/NF-κB signaling pathway (Choo et al., 2018; Wang et al., 2019), whereas M2-Exos contribute to tumor-associated immunosuppression (Mi et al., 2020). Drug-loaded M1-Exos have demonstrated synergistic effects in both targeted drug delivery and immune activation (Kim et al., 2016; Haney et al., 2020; Kim et al., 2018). However, large-scale production of these extracellular vesicles remains challenging, as the variability of macrophage-derived materials may affect batch-to-batch consistency and hinder clinical translation (Haney et al., 2020) (Figure 3).

### 4.6 Vascular-associated strategies targeting BAMs

BAMs, as important members of the neurovascular unit, play unique roles in regulating vascular integrity and inflammatory responses. In MS and experimental autoimmune encephalomyelitis (EAE) models, IGF-1 pathway inhibition selectively reprograms BAMs rather than microglia, thereby reducing CNS inflammation (Ivan et al., 2023); intracerebroventricular administration of RARα agonists can induce BRMs toward a neuroprotective phenotype (Ganz et al., 2024; Ganz et al., 2024). In a chronic progressive EAE model, even treatment initiated during the chronic stage improved clinical scores and prevented axonal loss, while inhibiting pro-inflammatory pathways and promoting microglia and BAMs toward a neuroprotective phenotype without affecting peripheral immune cell profiles (Ganz et al., 2024). Additionally, an open-label, crossover clinical study in 7 MS patients evaluated the safety and efficacy of recombinant human insulin-like growth factor-1 (rhIGF-1). The study showed that subcutaneous injection of rhIGF-1 (50 mg, bid) for 6 consecutive months did not significantly change MRI or clinical activity indicators, such as contrast-enhancing lesions, white matter lesion load, or T1 hypointense volume, but the drug was well tolerated without serious adverse effects. These results suggest that although rhIGF-1 monotherapy did not significantly improve disease progression, its good safety profile and potential mechanism in promoting oligodendrocyte differentiation and remyelination still provide clinical feasibility for future combination BAM-targeted therapies (Frank et al., 2002). Nevertheless, microglia-derived IGF-1 is also indicated a crucial supportive role in the proliferation and differentiation of GABAergic neuronal progenitors after IS (Yu et al., 2025). These findings indicate that direct targeting of CNS myeloid cells has potential neuroprotective value in neurological diseases and provides clinical feasibility for future BAM-targeted combination therapies.

### 4.7 Chemokine and oxidase inhibitors

Chemokine pathways participate in pro-inflammatory T cell recruitment and amplification of neuroinflammation. Microglia-derived CCL2/CCL8 mediates neuronal damage by activating CCR2/CCR5 receptors on circulating CD8^+^ T cells, exacerbating lesions in radiation-induced brain injury (RIBI) and ischemic stroke. Pharmacologic inhibition of CCL2/CCL8 or blocking T cell migration can reduce neuroinflammation, but receptor multi-specificity poses a high risk of off-target effects (Shi et al., 2023). Recent studies developed a NOX2-specific small molecule inhibitor, NCATS-SM7270, which selectively inhibits NOX2 in human and mouse granulocytes *in vitro* while reducing inhibition of xanthine oxidase. In a mild TBI (mTBI) mouse model, transcranial administration of NCATS-SM7270 dose-dependently reduced cortical cell death and partially reversed cortical damage in NOX4-deficient mice. This indicates that NOX2 plays a key role in mTBI pathology, and NCATS-SM7270 achieves neuroprotection through specific NOX2 inhibition (Mason et al., 2023).

### 4.8 Antibody and B cell-targeted therapies

B cell-depleting therapies (such as ocrelizumab, ofatumumab, ublituximab, and other monoclonal antibodies) can prevent B cells from entering the CNS, reducing pro-inflammatory interactions with microglia and alleviating pathological inflammation in MS (Yong and Yong, 2022; Jakimovski et al., 2024). Studies have shown that the B cell-depleting antibody ocrelizumab has been approved for the treatment of primary progressive MS (PPMS) with inflammatory activity, and the sphingosine-1-phosphate receptor modulator Siponimod is under development for secondary progressive MS (SPMS). These drugs target T cells, B cells, and microglia involved in chronic inflammatory responses, inhibiting reactive oxygen species release, reducing iron accumulation, and delaying neurodegeneration, providing new directions for treating progressive MS (Faissner and Gold, 2019). In the future, combining B cell inhibition with microglial reprogramming therapies may achieve multi-target immunomodulation to slow neuroinflammatory progression.

## 5 Perspectives

BRMs play dual roles in maintaining neural homeostasis and mediating neuroinflammation, providing new therapeutic opportunities for the precise modulation of neuroimmune functions. Accumulating evidence has revealed that microglia and BAMs act as both “executors” and “regulators” in neurodegenerative and inflammatory processes associated with AD, PD, MS, and brain injury. However, bridging the gap between basic mechanistic discoveries and clinical application requires achieving an optimal balance among target specificity, safety, and delivery efficiency.

Compared with cell-based therapies such as CAR-M, small molecules offer several advantages, including well-defined pharmacokinetics, reversibility, cost-effectiveness, and the ability to penetrate the BBB. Specific small-molecular regulators can be screened out through high-throughput screening and structural optimization to reprogram immune responses in disease loci. For instance, NLRP3 inflammasome inhibitors (such as DAPPD derivatives) can selectively block excessive inflammatory responses and reduce proinflammatory cytokine release at the source; TREM2 agonists enhance microglial phagocytic and metabolic activity, thereby restoring Aβ clearance and limiting neurotoxicity; and modulators targeting the ADGRG1–MYC signaling axis may promote lysosomal function and metabolic remodeling, optimizing the protective phenotype of microglia. Mechanistically, these compounds may achieve a “directed rebalance” of immune states, which selectively activates or suppresses key signaling nodes to shift BRMs from proinflammatory toward homeostatic or reparative phenotypes.

Small-molecular drugs can also be applied for targeting BAMs in cerebrovascular homeostasis and metabolic clearance. Targeting pathways such as CD36, SPP1, and ROS-related signaling may alleviate vascular oxidative stress and indirectly enhance cerebrospinal fluid circulation and the removal of toxic metabolites. Designing small-molecule inhibitors or receptor modulators that fine-tune these axes could restore the function of border immune compartments, achieving “outside-in” neuroprotection in neurological diseases. Moreover, innovative drug delivery systems are essential to achieving CNS-specific therapy. Advances in nanoparticles, liposomes, and BBB-penetrating chemically modified carriers offer promising solutions for the precision targeting and controlled release of small molecules. The integration of these delivery platforms with small-molecular modulators enables precise CNS intervention without the need for complex cell-engineering approaches. Through such approaches, BRMs may be transformed from “drivers of inflammation” into “mediators of neural repair”, opening new avenues for the pharmacological treatment of neurodegenerative diseases.
